# Pre-implantation exogenous progesterone and pregnancy in sheep: I. polyamines, nutrient transport, and progestamedins

**DOI:** 10.1186/s40104-021-00554-6

**Published:** 2021-03-05

**Authors:** Emily C. Hoskins, Katherine M. Halloran, Claire Stenhouse, Robyn M. Moses, Kathrin A. Dunlap, Michael C. Satterfield, Heewon Seo, Gregory A. Johnson, Guoyao Wu, Fuller W. Bazer

**Affiliations:** 1 Departments of Animal Science, Texas A&M University, College Station, TX 77843-2471 USA; 2 Veterinary Integrative Biosciences, Texas A&M University, College Station, TX 77843 USA

**Keywords:** Amino acid, Endometrium, Gene expression, Polyamine, Progesterone

## Abstract

**Background:**

Administration of exogenous progesterone (P4) to ewes during the pre-implantation period advances conceptus development and implantation. This study determined effects of exogenous P4 on transport of select nutrients and pathways that enhance conceptus development. Pregnant ewes (*n* = 38) were treated with either 25 mg P4 in 1 mL corn oil (P4, *n* = 18) or 1 mL corn oil alone (CO, *n* = 20) from day 1.5 through day 8 of pregnancy and hysterectomized on either day 9 or day 12 of pregnancy. Endometrial expression of genes encoding enzymes for synthesis of polyamines, transporters of glucose, arginine, and glycine, as well as progestamedins was determined by RT-qPCR.

**Results:**

On day 12 of pregnancy, conceptuses from P4-treated ewes had elongated while those from CO-treated ewes were spherical. The mRNA expression of *AZIN2,* an arginine decarboxylase, was lower in endometria of P4-treated than CO-treated ewes on day 9 of pregnancy. Expression of *FGF10*, a progestamedin, was greater in endometria of CO and P4-treated ewes on day 12 of gestation in addition to P4-treated ewes necropsied on day 9 of gestation. Treatment with P4 down-regulated endometrial expression of amino acid transporter *SLC1A4* on day 12 of pregnancy.

**Conclusions:**

Results indicated that administration of exogenous P4 during the pre-implantation period advanced the expression of *FGF10*, which may accelerate proliferation of trophectoderm cells, but also was correlated with decreased expression of glycine and serine transporters and polyamine synthesis enzyme *AZIN2.* Further research with increased sample sizes may determine how differential expression affects endometrial functions and potentially embryonic loss.

**Supplementary Information:**

The online version contains supplementary material available at 10.1186/s40104-021-00554-6.

## Background

Mechanisms responsible for establishment and maintenance of pregnancy in mammals are complex. Temporal and cell-specific regulation of expression of genes encoding for proteins is required for development of the conceptus (embryo and associated membranes), implantation, placentation, and vascularization of the uterus and conceptus for nutrient exchange [1–3]. This intricate process often fails as, of the 20–30% of embryonic death loss in sheep, other ruminants, and most mammalian species, two-thirds of these losses occur during the peri-implantation period [4–6]. Thus, research regarding embryonic mortality has focused on molecular mechanisms that positively influence conceptus development, pregnancy recognition signaling, implantation, and placentation. In sheep, the *corpus luteum* (CL) produces significant amounts of progesterone (P4) by day 3 of pregnancy and concentrations in maternal plasma increase to approximately 4 ng/mL on day 7 [7]. Uterine functions essential for the establishment and maintenance of pregnancy are regulated by P4, along with other hormones and growth factors, in all mammalian species studied to date. Ovine embryos enter the uterus as morulae on day 4 of pregnancy and by day 6, the uniform cells of the morula differentiate into the inner cell mass (ICM, precursors to the embryo/fetus) and trophectoderm (Tr) cells (precursors to the chorion of the placenta). Ovine blastocysts progress from approximately 200 μm in diameter on day 8 to between 400 and 900 μm by day 10, 10–22 mm by day 12, and 25 cm by day 17 of pregnancy [1, 8]. During elongation, ovine conceptuses secrete interferon tau (IFNT), the protein responsible for maternal recognition of pregnancy and maintenance of a functional CL to secrete progesterone (P4) [9–13]. P4 is required for successful establishment of pregnancy [14–17]; however, temporal- and cell-specific down-regulation of progesterone receptors (PGR) in the endometrial luminal epithelia (LE) and superficial glandular epithelia (sGE) is required for implantation and establishment of pregnancy [18]. Exogenous P4 therapy during the pre-implantation period of pregnancy accelerates growth and development of bovine and ovine conceptuses [19–23].

PGR are expressed in uterine LE between days 9 and 11, but down-regulation of PGR occurs after day 12 of gestation that alters the expression of genes in uterine epithelia that encode for secreted proteins and nutrient transporters responsible for accumulation of molecules, including nutrients, cytokines, and enzymes known collectively as histotroph [15, 17]. PGR expression is not down-regulated in uterine stromal cells (SC); therefore, P4 mediates it effects on uterine epithelia by inducing expression of growth factors known as progestamedins in uterine stromal cells [24]. Fibroblast growth factors 7 (FGF7) and FGF10, as well as hepatocyte growth factor (HGF) are known as progestamedins which bind to fibroblast growth factor receptor 2_IIIb_ and HGF receptors encoded by MET (a c-met proto-oncogene) on uterine LE, sGE, and conceptus Tr [25, 26]. The progestamedins act via their receptors in a paracrine fashion on uterine epithelial cells to stimulate secretion of histotroph and expression of transporters for nutrients. In sheep, *FGF10* mRNA is expressed by endometrial SC and mesenchymal cells of the chorioallantois, while *FGF7* is localized to endometrial and myometrial blood vessels [25]. HGF from uterine stromal cells mediates mesenchymal-epithelial cross-talk in the uterus and conceptus [27, 28].

Expression of the mRNA for the glucose transporter *SLC2A1* (solute carrier family 2 member 1) is up-regulated in ovine uterine LE and sGE by P4. This is further stimulated by IFNT and is accompanied by a six-fold increase of glucose in the uterine lumen between days 10 and 15 of pregnancy [29]. *SLC2A3* (solute carrier family 2 member 3), another glucose transporter, is expressed by ovine conceptus Tr and endoderm between days 12 and 20 of pregnancy [29]. Further, total amounts of glucose are greater in uterine flushings of ewes treated with exogenous P4 than those treated with corn oil (CO) during the pre-implantation period of pregnancy [30].

The gene *SLC5A1* (solute carrier family 5 member 1) encodes for the sodium dependent glucose transporter and its expression increases in uterine LE, sGE and GE between days 12 and 14 of gestation, but it is localized only to uterine GE between days 16 and 20 of pregnancy [29]. SLC5A1 protein is abundant on the apical surfaces of uterine LE between days 12 and 14 of pregnancy, indicating its importance for transport during the peri-implantation period of pregnancy. Further, its expression is greater in uterine LE and sGE of ewes treated with exogenous P4 in early pregnancy [29–31].

*SLC7A1*(solute carrier family 7 member 1) is a gene that encodes the System y^+^ high affinity cationic amino acid transporter. Arginine is a cationic amino acid of particular importance for implantation and pregnancy, as it can be metabolized to nitric oxide (NO) that stimulates angiogenesis and vasodilation of blood vessels, as well as polyamines utilized in many cellular functions required for conceptus development [32, 33]. Arginine plays a vital role during gestation, without which intrauterine growth restriction and altered genome expression of the fetus occurs [34]. In pregnant ewes, total amounts of arginine and histidine in uterine flushings increase 8- to 25-fold between days 10 and 16 of pregnancy [35, 36]. Treatment with exogenous P4 during the peri-implantation period of pregnancy increases the amount of arginine recovered from the uterine lumen on day 9 of pregnancy [30].

The gene *SLC6A9* (solute carrier family 6 member 9) encodes for the sodium and chloride dependent glycine transporter while *SLC1A4* (solute carrier family 1 member 4) encodes for an alanine/serine/cysteine/threonine transporter. Glycine and serine are the most abundant amino acids in uterine flushings from pregnant ewes [35]. Serine is the precursor for glycine that is metabolized to formate in one carbon metabolism, which is important for nucleotide synthesis. Further, serine affects protein synthesis, Ca^2+^ homeostasis, and the immune system, and acts as a neurotransmitter in the central nervous system [37]. There are no reports of expression of mRNAs for glycine or serine transporters by cells within the uterus of sheep during the peri-implantation period of pregnancy.

Polyamines (putrescine, spermidine, and spermine) and agmatine are essential for many cellular functions including proliferation, gene transcription and translation, angiogenesis, and antioxidants for reactive oxygen species [38]. Polyamines are derived from arginine in tissues and fluids of conceptuses during the peri-implantation and throughout the remainder of pregnancy in cattle, sheep, and pigs [34, 35]. Polyamines are essential for mammalian embryogenesis, implantation, and placentation [39–41]. Ovine conceptuses have the greatest amounts of polyamines, arginine, and ornithine on days 15 and 16 of pregnancy, the period of extensive elongation of the conceptus and preparation for adhesion of Tr cells to uterine LE. This is followed by differentiation of mononuclear trophoblast cells into trophoblast giant cells that will invade the uterine LE to form a syncytial layer [42, 43]. Treatment of ovine Tr cells with arginine increases their proliferation and production of IFNT through stimulation of the tuberous sclerosis complex 2 (TSC2)-mechanistic target of rapamycin (MTOR) cell signaling pathway [36]. Additionally, knock-down of translation of ornithine decarboxylase (*ODC1*) mRNA in ovine conceptuses led to the discovery of a secondary pathway for the conversion of arginine to polyamines via arginine decarboxylase (AZIN2) and agmatinase (AGMAT) enzymes [42]. Later, it was determined that all conceptuses failed to develop when translation of both *ODC1* and *AZIN2* (reported as *ADC* by Lenis et al. [44]) mRNAs were knocked down [44].

The present study, as the first in a set of two companion papers, examined specifically the effects of exogenous progesterone on the expression of major genes involved in the arginine-agmatine-polyamine pathway. Additionally, this study extends our previous knowledge on nutrient transport and molecular signaling during the peri-implantation period, and how exogenous P4 treatment affects these mechanisms responsible for advancing morphological development of ovine conceptuses.

## Methods

### Animals

Thirty-eight Suffolk mature ewes (*Ovis aries)* that had a minimum of two normal estrous cycles were placed with Suffolk rams (*n* = 5) of proven fertility when detected in estrus (day 0) for 36 h and rams were changed every 12 h. All experimental and surgical procedures were in compliance with and approved by Texas A&M University’s Guide for the Care and Use of Agriculture Animals in Research and Teaching.

### Experimental design and tissue collection

Bred ewes (*n* = 38) were assigned randomly to receive daily intramuscular injections of either 25 mg progesterone (P4, *n* = 18) in 1 mL corn oil vehicle or 1 mL corn alone (CO, *n* = 20) from day 1.5 through day 8 of pregnancy. Nine P4-treated ewes and 10 CO-treated ewes were euthanized and then hysterectomized on day 9 of pregnancy and 9 P4-treated ewes and 10 CO-treated ewes were euthanized and then hysterectomized on day 12 of pregnancy. Blood samples were taken from ewes via jugular venipuncture immediately prior to euthanasia and hysterectomy. Uterine flushings and blastocysts were collected for analyses by flushing the uterine horn with 10 mL of sterile phosphate buffered saline into a grid dish (pH = 7.2). The recovered volume of uterine flush was recorded. The volume of uterine flushings recovered for each group was not influenced by day or treatment (day 9 CO: 8.08 ± 0.08 mL; day 9 P4: 7.99 ± 0.47 mL; day 12 CO 8.06 ± 0.30 mL; and day 12 P4 8.43 ± 0.06 mL). Endometrial tissue from the uterine horn ipsilateral to the CL was collected and snap frozen in liquid nitrogen and stored at − 80 **°**C for quantitative real-time polymerase chain reaction (qPCR) analyses. Adjacent tissue was fixed in 4% paraformaldehyde for 24 h, transferred to 70% ethanol for 24 h, and then dehydrated through a graded series of alcohol to xylene and embedded in paraffin wax. Photomicroscopic images were taken using a Nikon SMZ18 camera for morphological analyses and volume measurements of all blastocysts and conceptuses in the grid dishes. Uterine flushes were centrifuged (5000 × *g* for 15 min at 4 °C) aliquoted into 1.5 mL tubes, and stored at − 20 °C until analyzed. Blood from ewes was stored on ice and centrifuged (10,000 × *g* for 7 min) to obtain plasma for radioimmunoassay. Plasma was aliquoted into 1.5 mL tubes and stored at − 20 °C until analyzed.

### Radioimmunoassay analysis for concentrations of progesterone in plasma

Concentrations of P4 in plasma were determined by using a Progesterone Coated Tube Radioimmunoassay Kit (07-270,102, MP Diagnostics) according to the manufacturer’s instructions and as previously described [45]. This radioimmunoassay was validated with P4 in ovine plasma.

### RNA isolation and quantitative real-time PCR analyses

Total RNA was isolated from endometria from the ipsilateral uterine horn of pregnant ewes with respect to CL using Trizol (Invitrogen) according to manufacturer’s instructions. Total RNA samples were cleaned using an RNeasy Mini Kit (Qiagen). RNA was quantified with a NanoDrop (ND-1000 Spectrophotometer) and quality was determined by spectrometry and bioanalysis (Bioanalyzer, Agilent). Samples with a RIN (RNA integrity number) greater than 7 were utilized to synthesize cDNA. Synthesis of cDNA from 5 μg total RNA was performed using the SuperScript™ First-Strand Synthesis System for qPCR (Invitrogen). Samples without reverse transcriptase were used as negative controls. Gene expression was analyzed via ABI PRISM 7700 (Applied Biosystems) with detector SYBR Green PCR Master Mix (Applied Biosystems) as described previously [26]. All primers (Supplemental Table 1) were designed utilizing NCBI Primer-Blast software. Efficiency of specificity of primers were determined via a standard curve created from pooled cDNA in addition to a dissociation curve for qPCR. Standards included serial dilutions of pooled cDNA in RNAse free water (Qiagen) from 1:2 to 1:256 dilution factor. Primers utilized produced a dissociation curve with one peak, signifying one single product. All chosen primers had an efficiency between 95% and 105%. For all primers excluding *AZIN2* and *AGMAT*, qPCR reactions were performed as described previously [42]. For primers of interest with lower expression (i.e. Cq values above 30; *AZIN2* and *AGMAT*), 1 μL of cDNA was used in a modified pre-amplification step [46] using a Thermocycler (Eppendorf AG). Briefly, cDNA, nuclease-free water, forward and reverse primer, and SYBR were combined in a 10-μL volume. The reaction was performed with the following conditions for 15 cycles: 94 °C for 30 s, 58 °C for 30 s, and 72 °C for 30 s. Tubulin was used as a reference gene as endometrial expression of tubulin was not affected by day or treatment. All mRNAs were calculated via the comparative Ct method [26].

### Immunohistochemistry

Paraffin-embedded sections (7 μm) were subjected to immunohistochemistry to determine cell-specific localization of ODC1, AZIN2, AGMAT, and PGR in endometria. Tissue processing and sectioning were completed as described previously [47]. Antigen retrieval was performed using boiling citrate for ODC1, AGMAT, and PGR and protease (0.5 mg/mL in phosphate buffered saline, PBS) for AZIN2. ODC1 protein was detected using a primary rabbit polyclonal antibody to ODC1 (Abcam, ab97395; Cambridge, UK) at a final dilution of 1:500. AZIN2 protein was detected using a primary rabbit polyclonal antibody (Abcam ab192771; Cambridge, UK) at a final dilution of 1:500. AGMAT protein was detected using a primary rabbit polyclonal antibody to AGMAT (Abcam 231,894; Cambridge, UK) at a final dilution of 1:250. Primary antibodies, excluding PGR, were replaced with rabbit IgG – Isotype at an equivalent protein concentration (Abcam; ab37355, Cambridge, UK) as a control. PGR protein was detected using a primary mouse monoclonal antibody against human PGR (Invitrogen; MA1-411, Carlsbad, CA, USA) at a final concentration of 1:500. PGR antibody was replaced with mouse IgG – Isotype at an equivalent protein concentration (Abcam, ab37355; Cambridge, UK). A universal rabbit and mouse Vectastain kit (Fisher Sci) was used for all proteins visualized in accordance with manufacturer instructions. Chromagen 3,3′-diaminobenzidine tetra-hydrochloride (Sigma) was utilized as the color substrate. Sections were counterstained with Harris hematoxylin (Sigma), dehydrated, and mounted with Permount (Fisher Sci). Imaging of slides was performed with a Nikon Eclipse Ni-U Microscope and NIS-Elements Software (Nikon) at 10× objective.

### Image analysis

All image analyses were performed using ImageJ. Stromal regions containing uterine glands and the luminal epithelial regions of the endometrium were analyzed separately. For analysis of endometrial sections stained for AGMAT and PGR, five non-overlapping images of stromal tissue containing uterine glands were taken at 10× magnification from each section. The images were split into red, green, and blue channels, and the percentage staining was quantified using the green channel at a threshold of 145- and 105-pixel intensity for AGMAT and PGR stained images respectively. For analysis of LE staining, three non-overlapping images were taken at 10× magnification of AGMAT, ODC, AZIN2, and PGR stained endometria from each section. The images were split into red, green, and blue channels and, using the freehand drawing tool on the green channel, the luminal epithelial area was selected, and the percentage staining was quantified [Threshold 145 (AGMAT) or 105 (ODC, AZIN2, and PGR) pixel intensity].

### Spectrophotometric assay

Glucose (Cell Biolabs Inc.) and fructose assay kits (Bioassay Systems, Enzychrom Fructose Assay Kit) were used to determine concentrations of glucose and fructose (nmol/mL), respectively in uterine flushings. The same glucose assay was used to determine the concentration of glucose in maternal plasma. Only uterine flushings and plasma from ewes which were considered pregnant with normally developed conceptuses were utilized for these analyses. Uterine flushings were diluted 1:2 for glucose and analyzed without dilution for the fructose assay. Maternal plasma was diluted 1:80 for quantification of glucose. Total recoverable glucose and fructose in uterine flushings was calculated by multiplying volume (mL) of uterine flush recovered by the concentration of glucose or fructose (nmol/mL). Late pregnancy samples from the companion papers to this study that had detectable levels of fructose were used as a positive control for samples in which fructose was undetectable (Halloran et al., unpublished results).

### HPLC analyses

Concentrations of amino acids and polyamines in uterine flushings and maternal plasma were determined via a high-performance liquid chromatography (HPLC) using a method described previously with some modifications [48]. Only uterine flushings and plasma from pregnant ewes were utilized for these analyses. Samples (100 μL) were acidified with 100 μLof 1.5 mol/L HClO_4_ and de-proteinized with 50 μL 2 mol/L K_2_CO_3_. The supernatant was diluted 1:10 and subjected to analysis by a precolumn derivatization *o*-phthaldialdehyde (OPA) reagent I or II HPLC method. For analysis of polyamines and agmatine, OPA reagent I was prepared by combining the following: 50 mg N-acetyl-cysteine (Sigma-Aldrich) 50 mg of OPA (Sigma-Aldrich) in 1.25 mL of HPLC-grade methanol (Fisher Scientific), 11.2 mL 0.04 mol/L sodium borate (pH 9.5), and 0.5 mL of Brij-23 (Sigma-Aldrich). For analysis of amino acids, OPA reagent II was prepared by combining the following: 50 mg OPA in 1.25 mL HPLC-grade methanol, 11.2 mL sodium borate (pH 9.5), 50 μL 2-mercaptoethanol, and 0.5 mL of Brij-23 (Sigma-Aldrich). The assay mixture consisted of 100 μL of sample, 1.4 mL of HPLC-grade water (Fisher Scientific), and 100 μL of 1.2% benzoic acid (in 40 mmol/L sodium borate, pH 9.5). The assay mixture was derivatized in an autosampler (model 712 WISP, Waters) using 30 mmol/L OPA reagent 1 or II before 15 μL of the derivatized mixture was injected into a Supelco 3-μm-reversed-phase C18 column (150 mm × 4.6 mm inner diameter, Sigma-Aldrich). Separation of amino acids, polyamines, and agmatine occurred using a solvent gradient consisting of solution A (0.1 mol/L sodium acetate, 18% methanol, and 1% tetrahydrofuran, pH 7.2) and solution B (methanol). All samples were quantified relative to authentic standards with Millenium-32 Software (Waters). Total amounts of amino acids, polyamines, and agmatine in maternal plasma and uterine flush were calculated by multiplying concentration by fluid volume.

### Statistical analysis

Data from the radioimmunoassay were analyzed via two-way analysis of variance (ANOVA) with ewe, treatment, and day as main effects and day by treatment as the interaction. Relative expression of mRNAs from qPCR analyses were analyzed using Proc GLM in SAS with data expressed as least square means (LSM) with standard errors of means (SEM) [49]. The probability of survival of blastocysts was determined by using the GLIMMX procedure in SAS with a maximum likelihood estimation technique. Data are represented as LSM ± SEM, with significant differences denoted by a different superscript letter. Statistical significance was set at *P* < 0.05) and trends for significance set at (*P* < 0.1).

## Results

### Concentrations of progesterone (P4) in maternal plasma

**Fig. 1:**
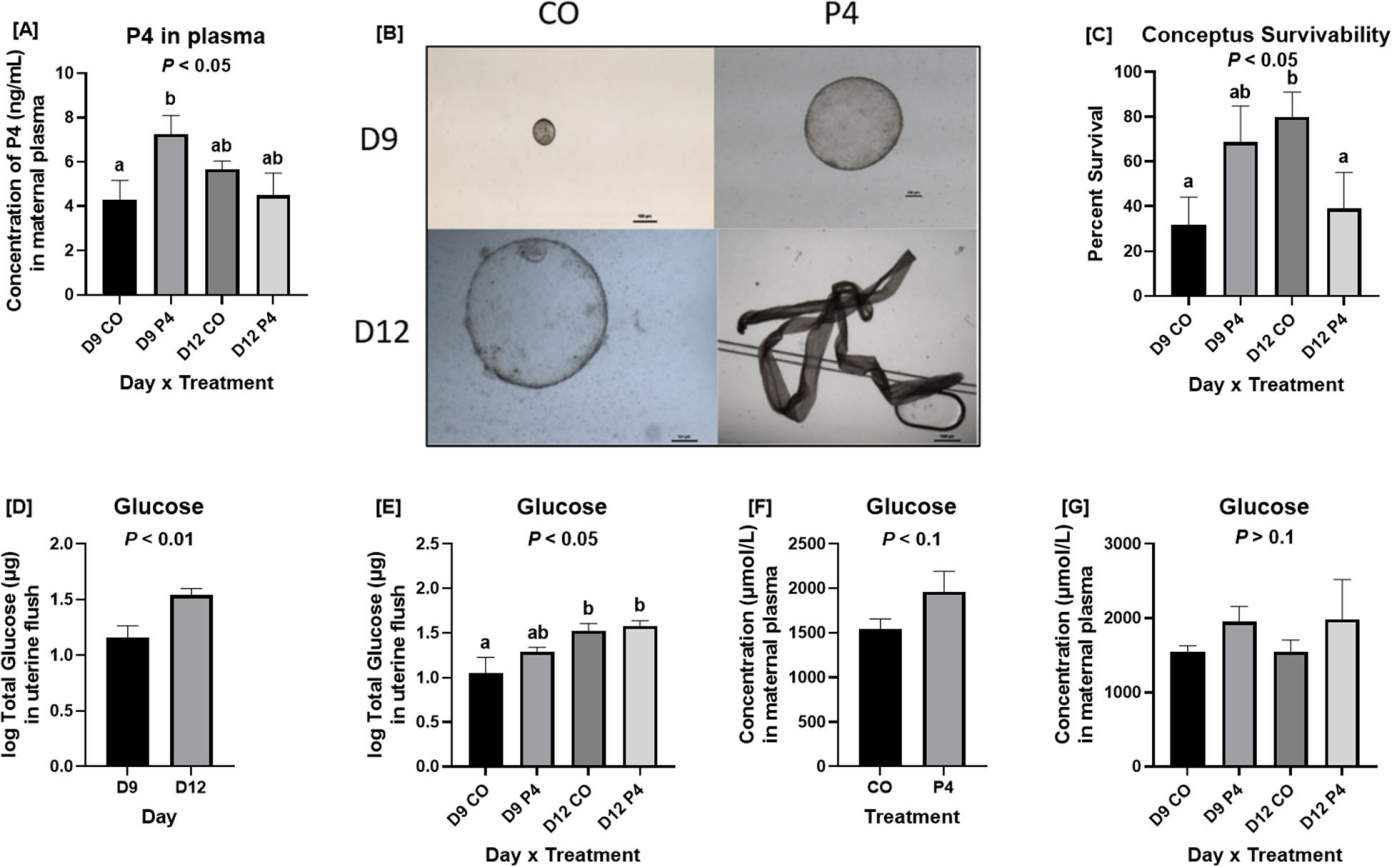
Effects of exogenous progesterone on blastocyst morphology, and plasma and uterine flushing composition. **a** Ewes necropsied on day 9 of pregnancy and treated with P4 had higher concentrations of P4 in plasma than CO-treated ewes (*P <* 0.05) but on day 12 concentrations of P4 in plasma were not different. There was a day × treatment interaction (*P <* 0.05). **b** All conceptuses from day 12 P4-treated ewes were filamentous while blastocysts from day 12 CO-treated ewes were spherical. The scale bar represents 100 μm for spherical blastocysts and 1000 μm for filamentous conceptuses. **c** Blastocysts from day 12 CO-treated ewes had a greater chance of surviving than those from day 12 P4-treated ewes (*P* < 0.05). There were no differences in the probability for survival of blastocysts from day 9 P4-treated and day 9 CO-treated ewes. There was a day × treatment interaction (*P* < 0.05). **d** Ewes necropsied on day 12 of pregnancy had greater concentrations of glucose in uterine flushings than ewes necropsied on day 9 of pregnancy (*P* < 0.01). **e** P4 and CO-treated ewes necropsied on day 12 of pregnancy had greater concentrations of glucose in uterine flushings than CO-treated ewes necropsied on day 9 of pregnancy (*P* < 0.05). **f** There was a trend for greater concentrations of glucose in the plasma of P4-treated ewes (*P* < 0.1). **g** Glucose concentrations in maternal plasma were affected by treatment but not day or their interaction. Data are presented as LSM ± SEM. Different means are indicated by different letters. Only uterine flushings and plasma from ewes which were considered pregnant with normally developed conceptuse were utilized for these analyses

A day × treatment interaction was identified for concentrations of P4 in maternal plasma (*P <* 0.05). Concentrations of P4 were greater in plasma of P4-treated than CO-treated ewes on day 9 of pregnancy, but on day 12 there was no difference in concentrations of P4 in plasma between P4- and CO-treated ewes (Fig.1a).

### Effects of exogenous P4 on blastocyst morphology, volume, and survival

Blastocysts from P4-treated ewes (0.025 mm^3^ ± 0.015 mm^3^) had, on average, greater volumes than blastocysts (0.013 mm^3^ ± 0.004 mm^3^) from control ewes on day 9, but differences in volumes were not statistically significant, presumably due to low sample size and natural variation. All conceptuses recovered from P4-treated ewes on day 12 were elongated and filamentous while conceptuses from CO-treated ewes were spherical (Fig. 1b). Ewes from which malformed blastocysts or unfertilized oocytes were recovered were considered not pregnant. Embryos which were arrested at the morula stage or that had ill-defined ICM and Tr based on quality standards described by the International Embryo Technology Society were considered malformed and not included in the study.

From the total 38 ewes bred, 6 day 9 P4-treated, 5 day 9 CO-treated, 4 day 12 P4-treated, and 9 day 12 CO-treated ewes were considered pregnant at the time of necropsy. A Maximum Likelihood test was performed using SAS to compare the number of blastocysts recovered with the number of CL present on the ovaries at necropsy (Fig. 1c). Blastocysts from day 12 CO-treated ewes had a greater chance of surviving than those from day 12 P4-treated ewes (*P <* 0.05). There were no differences in the probability for survival of blastocysts from day 9 P4-treated and day 9 CO-treated ewes (*P* > 0.05). There was a day × treatment interaction (*P* < 0.05).

### Effects of exogenous P4 on total glucose in uterine flushings

Total glucose in uterine flushings was affected by day (*P* < 0.01), and day × treatment interaction (*P* < 0.05) (Fig. 1d,e). Ewes necropsied on day 12 of pregnancy had greater concentrations of glucose in uterine flushings than ewes necropsied on day 9 of pregnancy (*P* < 0.01). P4 and CO-treated ewes necropsied on day 12 of pregnancy had greater concentrations of glucose in uterine flushings than CO-treated ewes necropsied on day 9 of pregnancy (*P* < 0.05). Glucose concentrations in maternal plasma were affected by treatment but not day or their interaction (Fig. 1f,g). There was a trend for greater concentrations of glucose in the plasma of P4-treated ewes (*P* < 0.1). Fructose was not detectable in uterine flushings from either day 9 or day 12 of pregnancy.

### Amino acids in maternal plasma and uterine flushings

Concentrations of amino acids in maternal plasma and uterine flushings are summarized in Tables 1 and 2, respectively. The most abundant amino acids in maternal plasma were glutamate, glycine, citrulline, arginine, alanine, valine, and leucine. There was a day × treatment interaction (*P* < 0.05) for aspartate in maternal plasma and concentrations of aspartate were greater in the plasma of P4-treated than CO-treated ewes on day 12. Concentrations of citrulline (*P* < 0.1) tended to be greater in the plasma of P4-treated than CO-treated ewes. Concentrations of aspartate were greater in the plasma of ewes on day 9 compared to day 12 of gestation (*P* < 0.05). In contrast, concentrations of β-alanine were greater in the plasma of ewes on day 12 than day 9 of gestation (*P* < 0.05).

**Table 1:** Concentrations of amino acids, agmatine, and polyamines in maternal plasma (nmol/mL)

	Effect			
	Day 9		Day 12	
Nutrient	CO	P4	CO	P4
Amino Acid				
Alanine	208.8 ± 26	170.7 ± 15.8	179.5 ± 12.9	207.4 ± 14.4
β-Alanine	7.9 ± 1.6^a^	7.9 ± 1^a^	10.5 ± 1.1^b^	9.9 ± 0.6^b^
Arginine	229.4 ± 32.1	168.5 ± 16.6	202.3 ± 13.5	206.1 ± 7.1
Asparagine	21.5 ± 4.5	23.2 ± 4.5	21.1 ± 3.1	19.9 ± 2.2
Aspartate	1.5 ± 0.4^ab^	2.6 ± 0.3^a^	0.8 ± 0.2^b^	1.6 ± 0.7^ab^
Citrulline	172.3 ± 20.4	198.2 ± 23	171.9 ± 28.6	246.8 ± 26.3
Glutamate	240.4 ± 17.2	236.5 ± 14.6	219.7 ± 14.2	226.6 ± 21.6
Glutamine	17.6 ± 5.2	21.5 ± 7.1	24.9 ± 3.9	32.5 ± 4.6
Glycine	384.6 ± 47.6	333 ± 65	322.9 ± 24.1	242.1 ± 22.5
Histidine	28.4 ± 3.5	28 ± 5	28.9 ± 2.8	32.4 ± 1.8
Isoleucine	82.9 ± 10.7	65.8 ± 4.3	66.3 ± 5	70.2 ± 7.9
Leucine	164.9 ± 26.3	164.3 ± 18.9	138.2 ± 13.7	164.1 ± 16.2
Lysine	115.5 ± 25.4	79 ± 7.1	91.1 ± 11.6	96.8 ± 18.2
Methionine	27.1 ± 5.5	24.8 ± 3	24.9 ± 2.8	23.1 ± 2.1
Ornithine	99.5 ± 33.3	102.4 ± 20.3	83.5 ± 12.7	119.2 ± 33.6
Phenylalanine	51.1 ± 5.8	54.7 ± 4.9	46.2 ± 6.2	48.6 ± 2.3
Serine	70 ± 8.3	53.3 ± 14.4	54.6 ± 7.5	51.7 ± 5.1
Taurine	78.5 ± 23.1	86.8 ± 17	68.7 ± 12.2	72.8 ± 7.6
Threonine	95.3 ± 20.9	110.8 ± 29.2	89.7 ± 16.1	92.1 ± 10.4
Tryptophan	41.6 ± 6.8	43.6 ± 2.1	41.6 ± 4.3	43.8 ± 2.2
Tyrosine	66.2 ± 8.6	69.5 ± 5.6	64.1 ± 4.9	69 ± 8.7
Valine	198.7 ± 30.3	224.8 ± 18.7	185.7 ± 19.3	215.1 ± 23.3
Agmatine and polyamines				
Agmatine	54.2 ± 9.6	53.6 ± 5.8	57.6 ± 2.9	57.54 ± 3.5
Putrescine	1.5 ± 0.4	1.1 ± 0.2	1.3 ± 0.1	1.3 ± 0.3
Spermidine	2.5 ± 0.1^ab^	2.9 ± 0.2^a^	2.3 ± 0.1^b^	2.5 ± 0.1^ab^
Spermine	4.4 ± 0.4^a^	6.2 ± 0.7^a^	2.7 ± 0.1^b^	3.3 ± 0.1^bc^

Values represent means ± SEM

Different superscripts within Effect columns are significantly different (*P* < 0.05)

Only maternal plasma from ewes which were considered pregnant with normally developed conceptuse were utilized for these analyses

**Table 2:** Total amino acids, agmatine, and polyamines in uterine flushings (μg)

	Effect			
	Day 9		Day 12	
Nutrient	CO	P4	CO	P4
Amino Acid				
Alanine	5 ± 1.5^a^	7.2 ± 1.9^a^	8.6 ± 2.2^a^	17.8 ± 1.4^b^
β-Alanine	0.2 ± 0.04	0.4 ± 0.2	0.4 ± 0.1	0.5 ± 0.2
Arginine	2.5 ± 1.3	4.1 ± 1.8	7.2 ± 2.2	8.3 ± 3.8
Asparagine	1.5 ± 0.5^a^	1.8 ± 0.6^ab^	4.2 ± 1^ab^	5.2 ± 0.6^b^
Aspartate	2.2 ± 0.5^ab^	1.6 ± 0.3^a^	4.1 ± 0.8^b^	4.2 ± 0.5^ab^
Citrulline	0.7 ± 0.2	3.4 ± 1.6	3.7 ± 1.1	1.8 ± 1.2
Glutamate	17.5 ± 3.6^ab^	8.8 ± 1.3^a^	23.8 ± 3.5^b^	29.1 ± 3.5^b^
Glutamine	3.1 ± 1	3.7 ± 1.5	11.5 ± 2.7	12.1 ± 2.3
Glycine	65.6 ± 21.4	81.5 ± 16.4	76.6 ± 14.8	59 ± 4.8
Histidine	0.8 ± 0.4	1.4 ± 0.6	2.1 ± 0.5	2.4 ± 0.9
Isoleucine	2 ± 1.8^ab^	1.5 ± 0.2^a^	1.5 ± 0.2^a^	3.1 ± 0.4^b^
Leucine	6 ± 1.8	3.7 ± 1.1	6.6 ± 1.2	9.1 ± 1.2
Lysine	6 ± 2	2.8 ± 0.6	5.2 ± 1.5	10.9 ± 2.7
Methionine	1.4 ± 0.6	1.1 ± 0.5	2 ± 0.6	3.4 ± 1
Ornithine	0.2 ± 0.06^a^	0.4 ± 0.1^ab^	0.5 ± 0.1^ab^	0.9 ± 0.2^b^
Phenylalanine	2.7 ± 0.9	1.9 ± 0.5	1.9 ± 0.4	3.5 ± 1
Serine	12 ± 3.6^a^	41.1 ± 12.2^b^	41.4 ± 10.1^b^	52.9 ± 13.1^b^
Taurine	20 ± 8.3	18.3 ± 4.4	23 ± 5.7	25.5 ± 0.7
Threonine	3.4 ± 1.4	6.7 ± 1.7	13.4 ± 3.8	12.4 ± 3.9
Tryptophan	0.7 ± 0.4	1.3 ± 0.4	1.6 ± 0.3	1.4 ± 0.7
Tyrosine	1.7 ± 0.8	1.3 ± 0.4	1.2 ± 0.2	2.9 ± 1.3
Valine	10 ± 3.4	5.6 ± 1.4	6.1 ± 1.1	5.8 ± 0.6
Agmatine and polyamines				
Agmatine	0.1 ± 0.04	0.04 ± 0.01	0.08 ± 0.02	0.02 ± 0.0001
Putrescine	1.1 ± 0.5	0.8 ± 0.4	1.1 ± 0.3	0.1 ± 0.03
Spermidine	0.9 ± 0.4	0.8 ± 0.4	1.3 ± 0.3	0.1 ± 0.03
Spermine	1.2 ± 0.6	2 ± 1.2	2.3 ± 0.7	1.1 ± 0.8

Values represent means ± SEM

Different superscripts within Effect columns are significantly different (*P* < 0.05)

Only uterine flushings from ewes which were considered pregnant with normally developed conceptuses were utilized for these analyses

Glutamate, serine, glycine, and taurine were the most abundant amino acids in uterine flushings. A day × treatment interaction was observed for concentrations of aspartate (*P* < 0.05), glutamate (*P <* 0.01), asparagine (*P* < 0.05), serine (*P* < 0.05), and isoleucine (*P* < 0.05). Concentrations of aspartate (*P* < 0.001), glutamate (*P* < 0.01), asparagine (*P* < 0.01), glutamine (*P* < 0.01), threonine (*P* < 0.05), alanine (*P* < 0.05), and ornithine (*P* < 0.01) were all greater in uterine flushings from ewes on day 12 than day 9 of pregnancy. Concentrations of serine, histidine, arginine, methionine, and leucine all tended to be greater in uterine flushings from ewes on day 12 than day 9 of pregnancy (*P* < 0.1). Concentrations of glutamate were less in the uterine flushings of P4-treated ewes on day 9 of pregnancy than CO-treated and P4-treated ewes on day 12 of pregnancy (*P* < 0.01). Concentrations of serine in uterine flushings were greater in P4-treated ewes on day 9 and 12 of pregnancy and CO-treated ewes necropsied on day 12 of pregnancy than in CO-treated ewes necropsied on day 9 of pregnancy (*P* < 0.05). Concentrations of isoleucine were greater in uterine flushings from P4-treated ewes than for CO-treated ewes on day 12 of gestation (*P* < 0.05). Serine was more abundant in uterine flushings from P4-treated than CO-treated ewes necropsied on day 9 of pregnancy (*P* < 0.05).

### Agmatine and polyamines in maternal plasma and uterine flushings

**Fig. 2:**
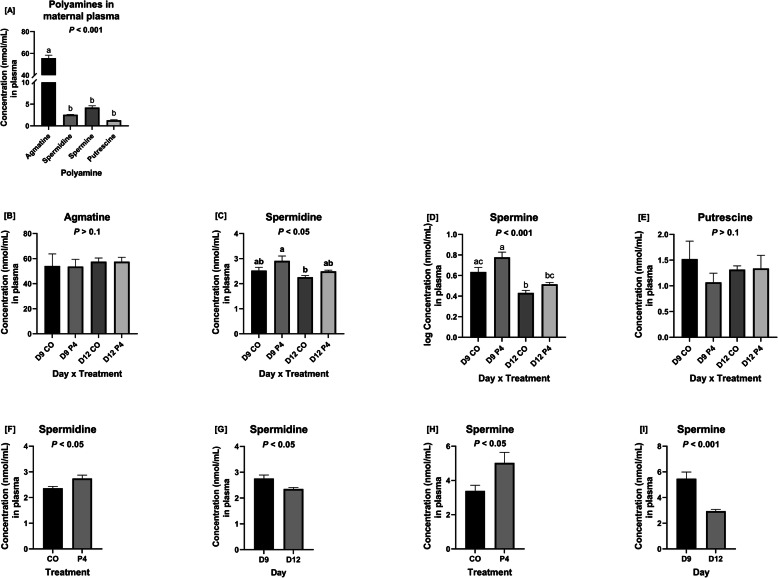
Concentrations of agmatine and polyamines in maternal plasma. **a** Agmatine was more abundant than any of the polyamines in maternal plasma (*P* < 0.001). **b** There was not a significant day × treatment interaction for concentrations of agmatine in maternal plasma. **c** Concentrations of spermidine in maternal plasma were affected by a day × treatment interaction (*P* < 0.05). **d** Concentrations of spermine in maternal plasma were affected by a day × treatment interaction (*P* < 0.001). **e** Concentrations of putrescine in maternal plasma were not affected by a day × treatment interaction. **f** P4-treated ewes had lower concentrations of spermidine in maternal plasma than CO-treated ewes (*P* < 0.05). **g** Ewes on day 12 of gestation had decreased amounts of spermidine in plasma than ewes on day 9 of gestation. **h** P4-treated ewes had greater amounts of spermine in their plasma than CO-treated ewes (*P* < 0.05). **i** Ewes on day 12 of pregnancy had decreased amounts of spermine in maternal plasma than ewes on day 9 of pregnancy (*P* < 0.01). Only plasma from ewes which were considered pregnant with normally developed conceptuses were utilized for these analyses

Overall, agmatine was more abundant than any of the polyamines in maternal plasma (*P* < 0.001) (Fig. 2a). In maternal plasma, concentrations of spermidine (*P* < 0.05) and spermine (*P* < 0.001) were affected by a day × treatment interaction (Fig. 2c,d). Concentrations of spermidine and spermine were greater for P4-treated compared to CO-treated ewes (*P* < 0.05) (Fig. 2f,h) while concentrations of spermidine (*P* < 0.05) and spermine (*P* < 0.001) were greater on day 9 than day 12 of pregnancy (Fig. 2g,i).

**Fig. 3:**
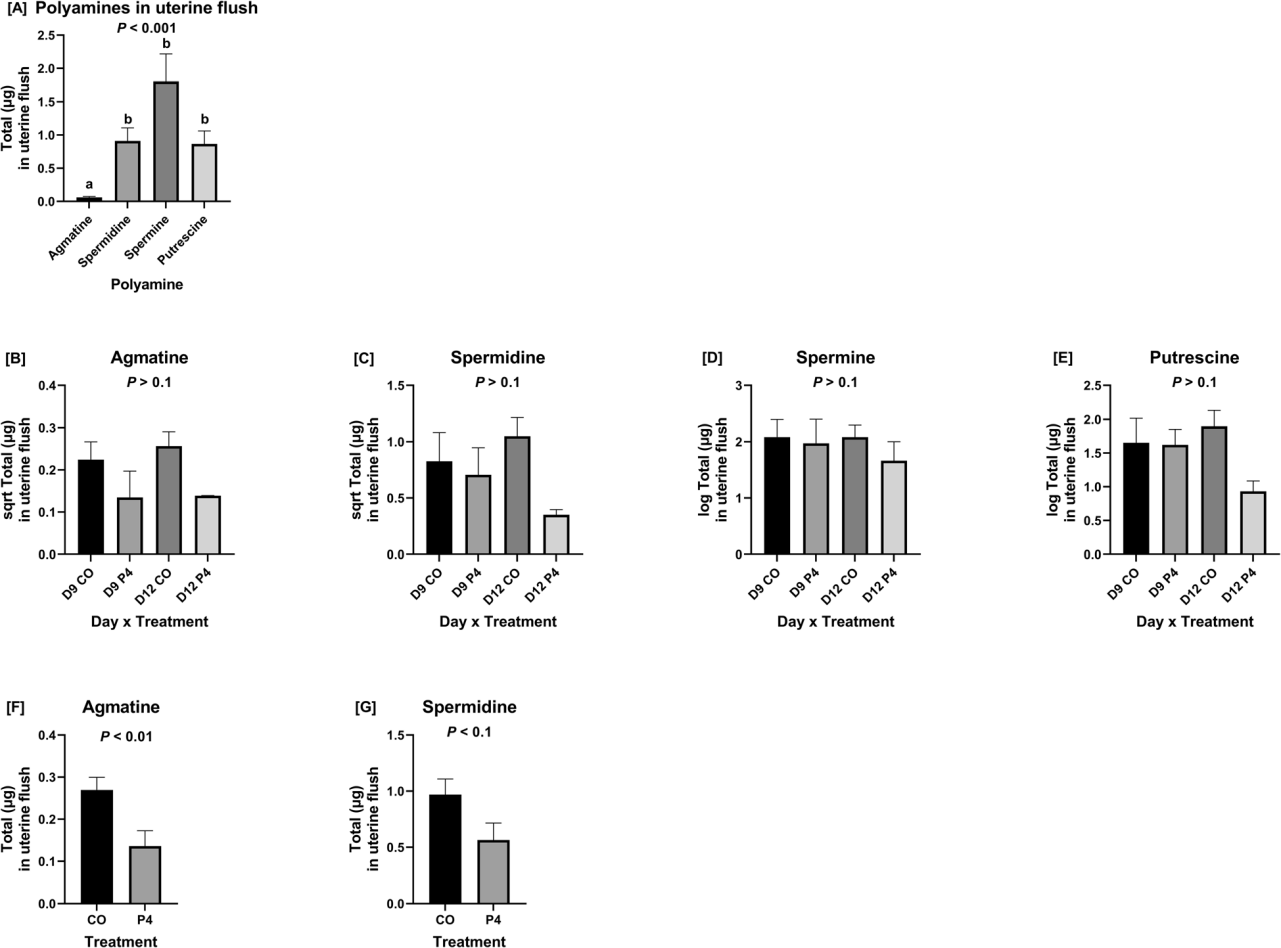
Concentrations of agmatine and polyamines in uterine flushings. **a** Agmatine was less abundant than any of the polyamines in uterine flushings. **b** Concentrations of agmatine in uterine flushings were not affected by a day × treatment interaction. **c** Concentrations of spermidine in uterine flushings were unaffected by a day × treatment interaction. **d** Concentrations of spermine in uterine flushings were not affected by a day × treatment interaction. **e** Concentrations of putrescine in uterine flushings were not affected by a day × treatment interaction. **f** P4-treated ewes had lower concentrations of agmatine in uterine flushings than CO-treated ewes (*P* < 0.01). **g** P4-treated ewes tended to have lower concentrations of spermidine in uterine flushings than CO-treated ewes (*P* < 0.1). Only uterine flushings from ewes which were considered pregnant with normally developed conceptuses were utilized for these analyses

Interestingly, agmatine was less abundant than all other polyamines in uterine flushings from ewes in all treatment groups (Fig. 3a). Concentrations of agmatine in uterine flushings were less in those from P4-treated than CO-treated ewes (*P* < 0.01) and concentrations of spermidine tended to be less in the uterine flushings from P4-treated than CO-treated ewes (*P* < 0.1) (Fig. 3f,g). There was no effect of day of pregnancy on abundances of agmatine or polyamines in uterine flushings (Fig. 3b,c,d,e).

### Expression of mRNAs in the endometrium

**Fig. 4:**
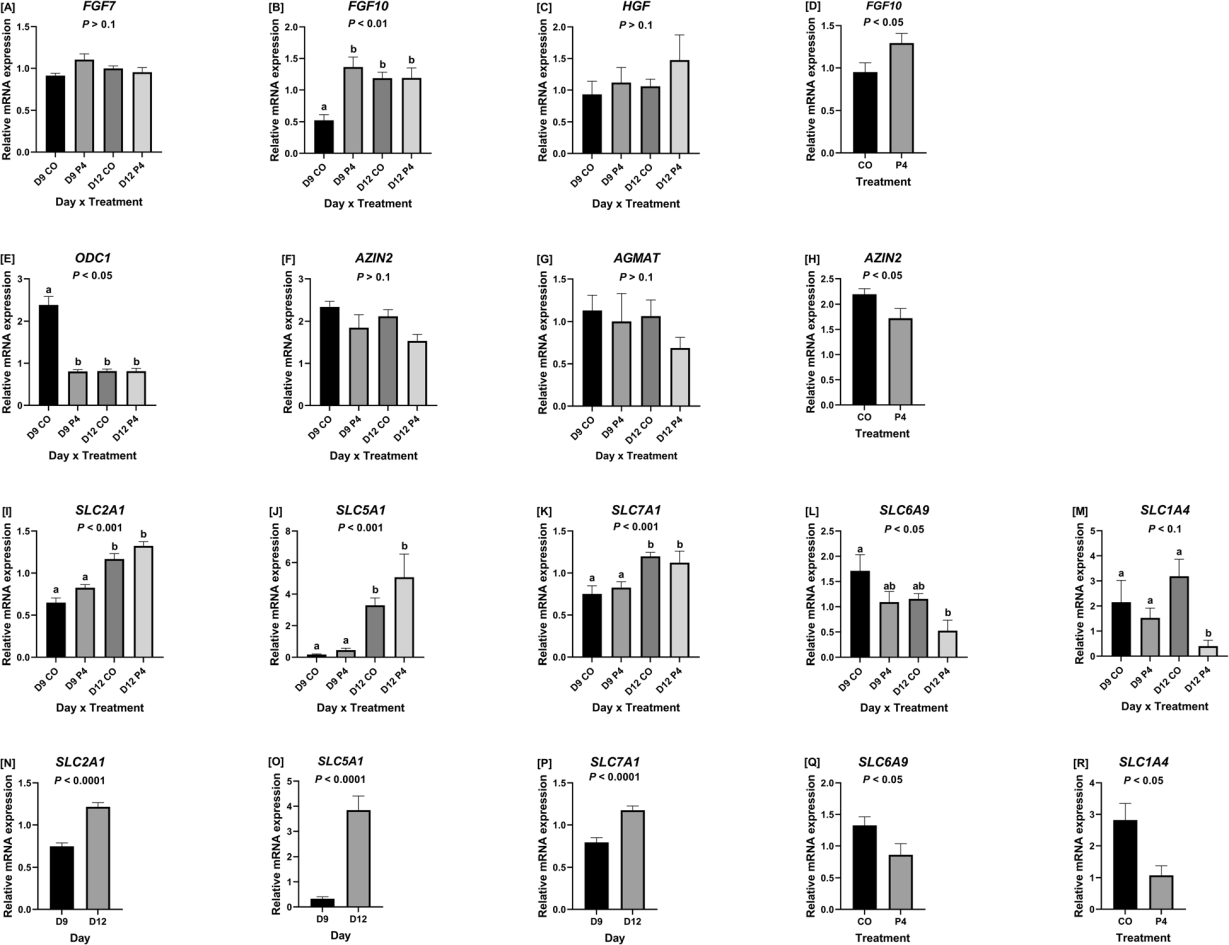
Endometrial expression of mRNAs for progestamedins, enzymes involved in polyamine synthesis, and nutrient transporters. Expression of *FGF10* (**d**), *AZIN2* (H), *SLC6A9* (**q**), and *SLC1A4* (**r**) were affected by treatment. Expression of *SLC2A1* (**n**), *SLC5A1* (**o**), and *SLC7A1* (**p**) were affected by day. Expression of *FGF10* (**b**), *ODC1* (**e**), *SLC2A1* (**i**), *SLC5A1* (**j**), *SLC7A1* (**k**), and *SLC6A9* (**l**) were affected by a day × treatment interaction. Expression of *FGF7* (**a**), *HGF* (**c**), *AZIN2* (**f**)*,* and *AGMAT* (**g**) were not affected by a day × treatment interaction. There was a tendency for the endometrial expression of *SLC1A4* (**m**) to be affected by a day × treatment interaction. Data are presented as LSM ± SEM. Different means are indicated by different letters. Only endometria from ewes which were considered pregnant with normally developed conceptuse were utilized for these analyses

Expression of *SLC2A1* (facilitated glucose transporter) mRNA was greater (*P* < 0.001) in the endometria of ewes on day 12 of pregnancy, and there was a day × treatment interaction (*P <* 0.001), but no effect of treatment was observed (Fig. 4i,n). Expression of *SLC5A1* mRNA (glucose and sodium co-transporter) was greater on day 12 than day 9 of pregnancy (*P* < 0.001) and expression was affected by a day × treatment interaction (*P <* 0.001), but not treatment (Fig. 4j,o). Similarly, expression of *SLC7A1* mRNA (cationic amino acid transporter) was affected by day (*P* < 0.001) and a day × treatment interaction (*P <* 0.001), but not treatment (Fig. 4k,p). Ewes on day 12 of pregnancy had greater expression of endometrial *SLC7A1* mRNA than ewes necropsied on day 9 of pregnancy.

There was no treatment, day, or day × treatment interaction for expression of *FGF7* mRNA in endometria (Fig. 4a). However, expression of *FGF10* was affected by treatment (*P <* 0.05) and a day × treatment interaction (*P* < 0.01), but not day (Fig. 4b,d). CO-treated ewes on day 12 of pregnancy and P4-treated ewes on day 9 and day 12 of pregnancy had greater (*P* < 0.05) expression of *FGF10* mRNA in their endometria than CO-treated ewes on day 9 of pregnancy. P4-treated ewes had greater (*P* < 0.05) expression of *FGF10* mRNA in their endometria than CO-treated ewes. Expression of *HGF* in endometria was not affected by treatment, day or their interaction (Fig. 4c).

*SLC6A9* mRNA (sodium- and chloride-dependent glycine transporter 1) endometrial expression was affected by treatment (*P* < 0.05) and day × treatment interaction (*P* < 0.05) but not day (Fig. 4l,q). Expression of *SLC1A4* mRNA (neutral amino acid transporter for amino acids including serine) in endometria was affected by treatment (*P* < 0.05) and a day × treatment interaction (*P* = 0.053), but not day of pregnancy (Fig. 4m,r). Endometria from P4-treated ewes on d 12 of gestation expressed less *SLC1A4* than CO-treated ewes on days 9 and 12 of gestation and P4-treated ewes on day 9 of gestation (*P* = 0.053). Overall, CO-treated ewes had greater (*P* < 0.05) expression of both *SLC6A9* and *SLC1A4* than P4-treated ewes.

Endometrial expression of *ODC1* mRNA was affected by a day × treatment interaction (*P* < 0.05), but not day of gestation or treatment (Fig. 4e). CO-treated ewes on day 9 of pregnancy had greater expression of *ODC1* mRNA than P4-treated ewes on either day 9 or day 12 of pregnancy and CO-treated ewes on day 12 of pregnancy (*P* < 0.05). Endometrial expression of *AZIN2* mRNA was affected by treatment (*P* < 0.05), but not by a day or day × treatment interaction (Fig. 4f,h). Expression of *AZIN2* mRNA was less for P4-treated than CO-treated ewes. Expression of AGMAT was unaffected by treatment, day, or day × treatment interaction (Fig. 4g).

### Localization of enzymes associated with synthesis of polyamines in endometria

**Fig. 5:**
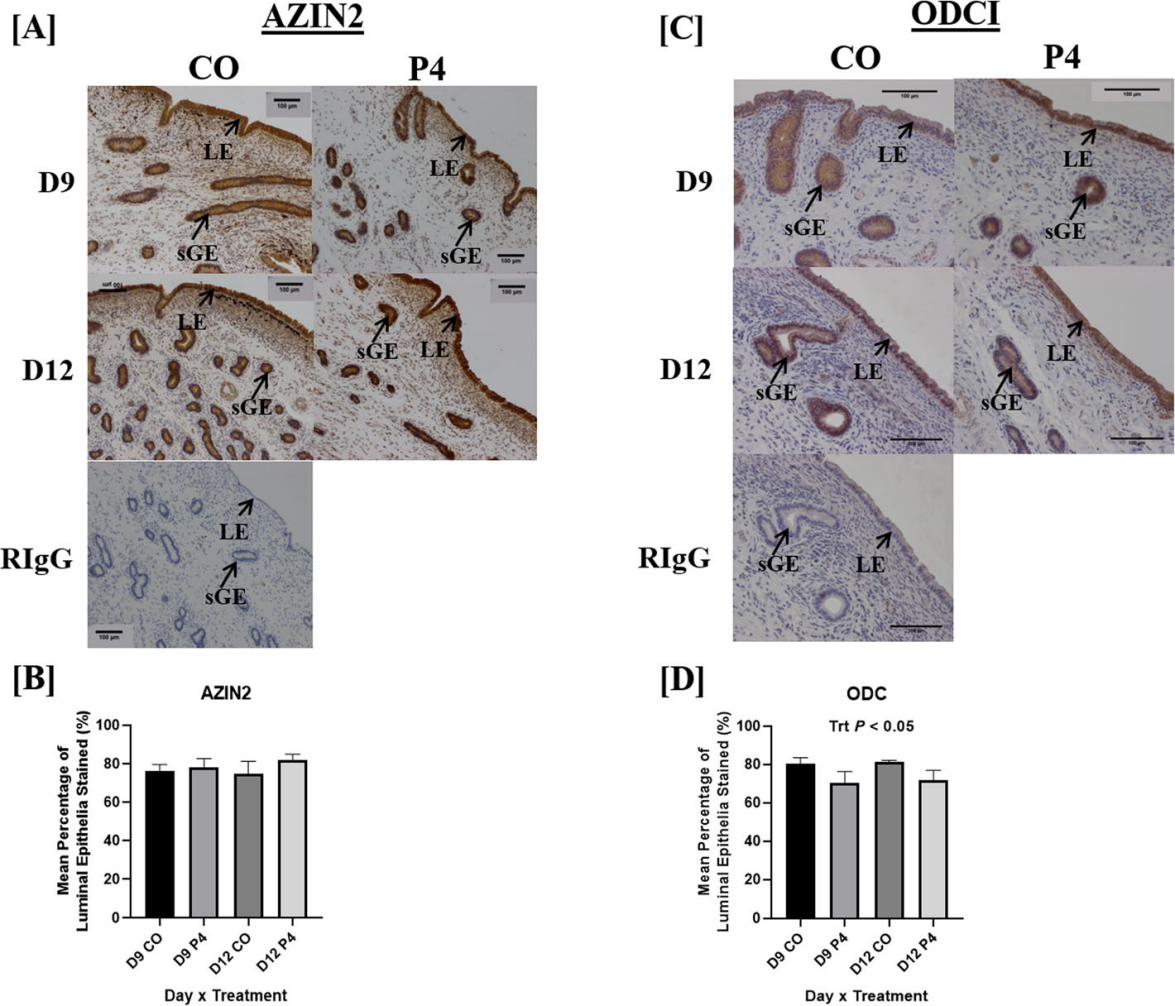
Localization of endometrial enzymes involved in polyamine synthesis. AZIN2 and ODC1 proteins localized to uterine luminal epithelia (LE), superficial glandular epithelia (sGE), and stromal cells in both P4- and CO-treated ewes on day 9 and 12 of pregnancy (**a** and **c**). Intensity of AZIN2 staining in the LE was not associated with day or treatment (**b**). Intensity of ODC1 staining in the LE was decreased in endometria from P4-treated ewes compared to CO-treated ewes (**d**). Scale bar represents 100 μm. Only endometria from ewes which were considered pregnant with normally developed conceptuses were utilized for these analyses

**Fig. 6:**
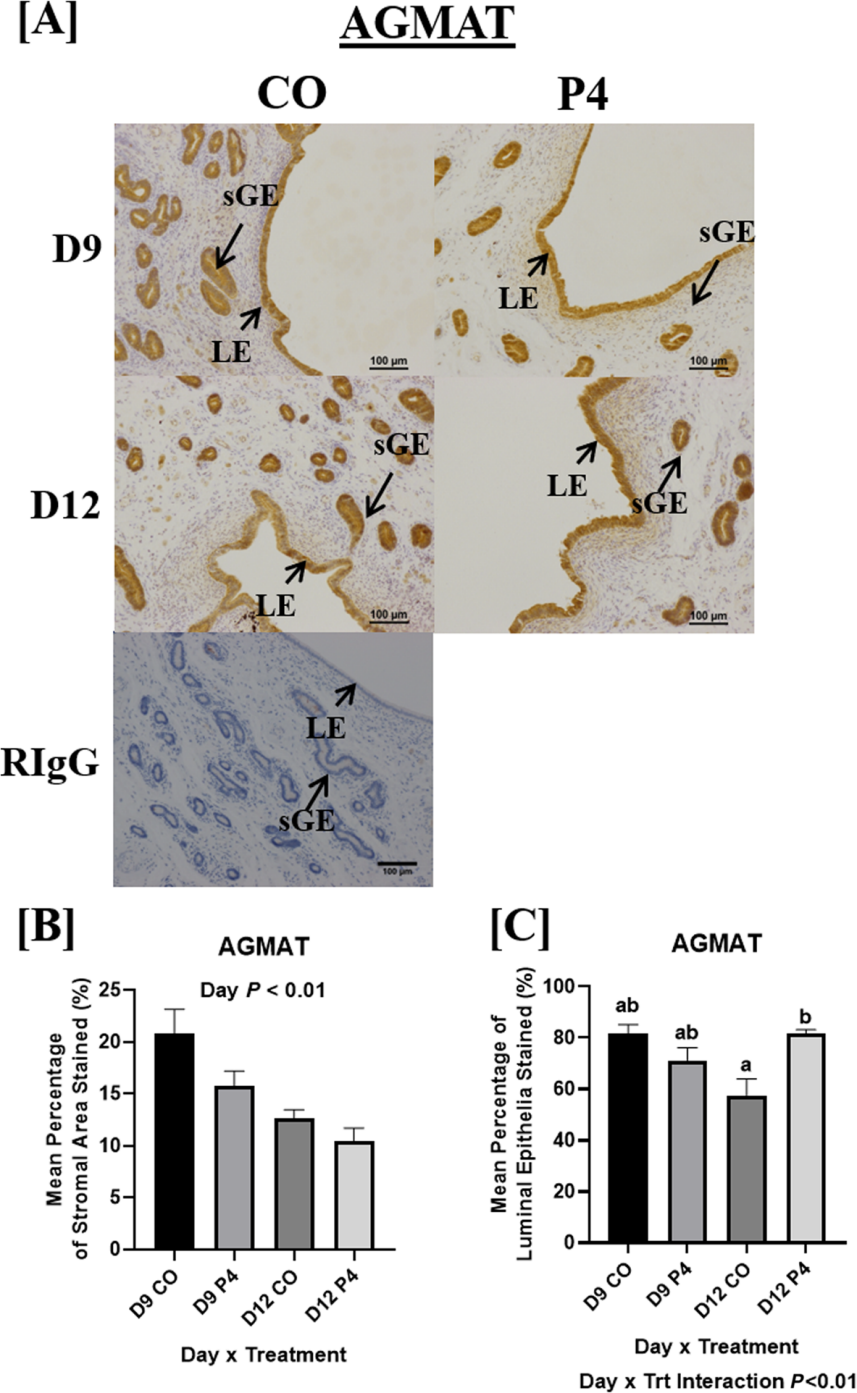
Localization of endometrial AGMAT. AGMAT proteins localized to uterine luminal epithelia (LE), superficial glandular epithelia (sGE), GE, and stromal cells in both P4- and CO-treated ewes on day 9 and 12 of pregnancy (**a**). Intensity of AGMAT staining in the GE was decreased in endometria at day 12 compared to day 9 (*P* < 0.01) (**b**). A day × treatment interaction for the intensity of AGMAT staining in the LE was observed (*P* < 0.001), with increased intensity of AGMAT staining observed in the LE from P4-treated ewes compared to CO-treated ewes at day 12. Scale bar represents 100 μm. Only endometria from ewes which were considered pregnant with normally developed conceptuses were utilized for these analyses

AZIN2 and ODC1 (Fig. 5) proteins localized to uterine LE, sGE, and stromal cells in both P4- and CO-treated ewes. AZIN2 staining intensity in LE was not altered by day or treatment (Fig. 5a and b). In contrast, a decrease in ODC1 staining intensity was observed in P4-treated ewes compared to CO-treated ewes (Fig. 5c and d). AGMAT protein localized to the uterine LE, sGE, GE, and stromal cells in both P4- and CO-treated ewes (Fig. 6). Decreased intensity of AGMAT staining was observed in the GE of ewes necropsied on day 12 of gestation compared to day 9 (Fig. 6a and b). Interestingly, a day × treatment interaction was observed for AGMAT staining in the LE, with LE in endometria from P4-treated ewes having increased intensity of AGMAT staining compared to CO-treated ewes necropsied on day 12 of gestation (Fig. 6a and c).

**Fig. 7:**
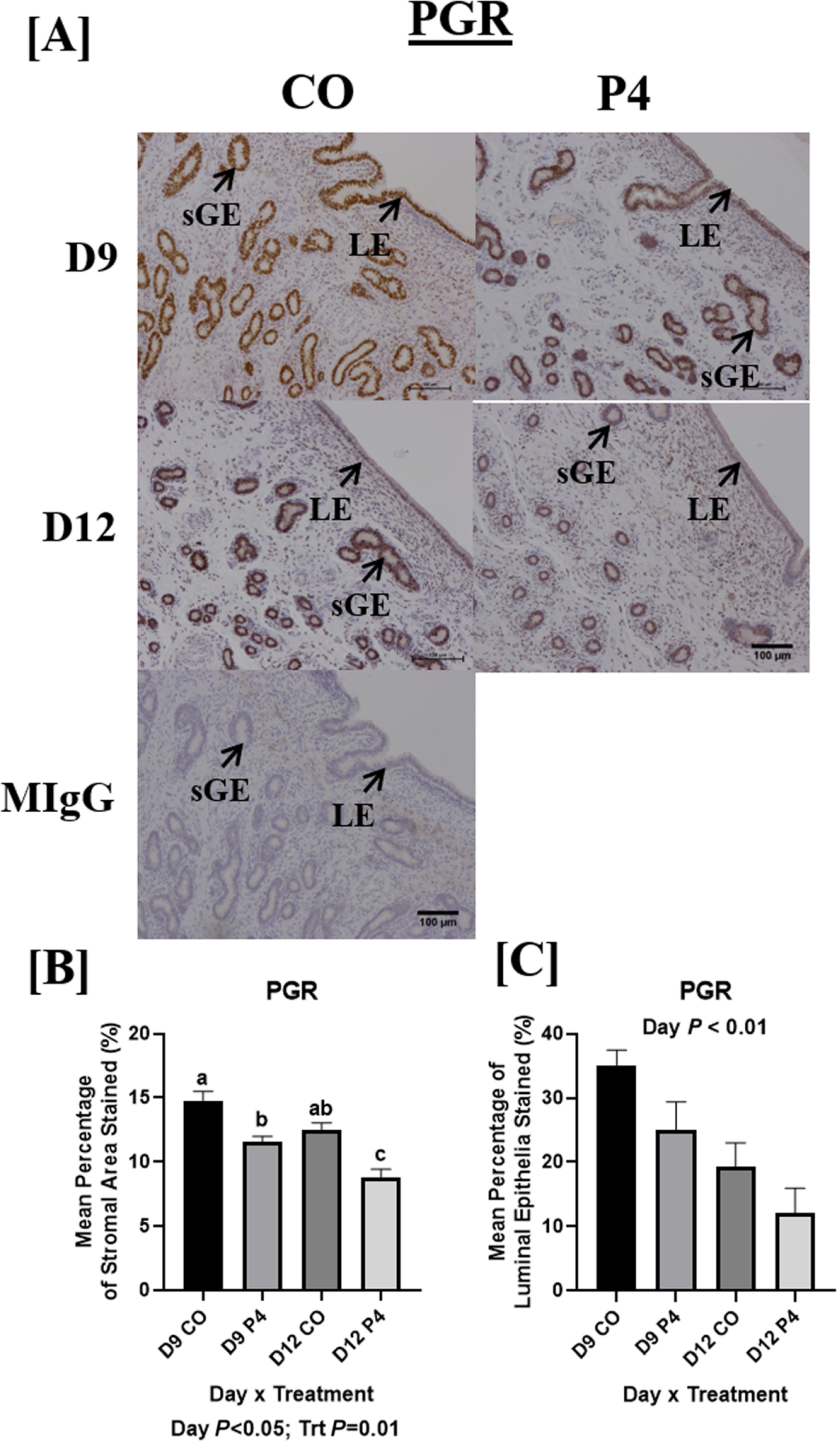
Localization of endometrial PGR. PGR staining was most abundant in endometrial luminal epithelium (LE) and superficial glandular epithelia (sGE) in CO-treated ewes necropsied on day 9 of pregnancy and was markedly reduced in endometria of P4-treated ewes necropsied on day 9 and 12 (**a** and **b**). Similar PGR localization and staining intensity was found in the LE of CO and P4-treated ewes necropsied on day 12. PGR in endometria of P4-treated ewes necropsied on day 12 was least abundant in both sGE and LE. Scale bar represents 100 μm. Only endometria from ewes which were considered pregnant with normally developed conceptuse were utilized for these analyses

PGR protein was localized to endometrial LE, sGE, and GE, with differences in staining intensity and localization observed depending on treatment and day (Fig. 7). PGR staining was less intense in ewes necropsied on day 12 of gestation compared to day 9 of gestation (Fig. 7b and c). Increased intensity of PGR staining was observed in the GE (Fig. 7a and b) in control ewes compared to P4-treated ewes necropsied on day 9 and 12. Whilst a significant decrease in the intensity of PGR staining in GE was observed in P4-treated ewes compared to CO-treated ewes necropsied on day 12 (Fig. 7a), LE expression of PGR had similar localization and intensity between CO- and P4-treated ewes.

## Discussion

In the present study, exogenous P4 given to pregnant ewes from days 1.5 to 8 of pregnancy resulted in elongated, filamentous conceptuses while control ewes administered corn oil had spherical blastocysts on day 12 of pregnancy. These results confirm previous findings and clearly demonstrate that an early increase in concentrations of P4 induce changes in endometrial functions that accelerate development of the conceptus during the peri-implantation stages of development. Of particular interest is the discovery that treatment with P4 and/or day of gestation alters some key metabolic parameters, including amino acids in the maternal plasma, uterine flushings, and the expression of multiple genes in the endometrium. Previous research demonstrated that administration of exogenous P4 during the pre-implantation period of pregnancy advances growth and development of bovine and ovine conceptuses [21, 30, 31]. This phenomenon has previously been attributed to the advancement of the down-regulation of PGR in endometrial sGE and LE [22]. Without exogenous progesterone treatment, PGR is present from day 9 to day 11 [18]. In the present study, immunohistochemical localization of PGR confirmed that advancement of PGR down-regulation occurred in the present study. It is important to note that in addition to down-regulation of PGR, the ewes and conceptuses would also be subjected to a decrease in P4 following cessation of P4 treatment. Considering this, there may be other indirect effects of P4 on both the conceptus and the endometrium which warrant further investigation.

This study suggests that blastocysts from day 12 P4-treated ewes were less likely to survive than blastocysts from day 12 CO-treated ewes and blastocysts from P4- and CO-treated ewes had equivalent rates of survival on day 9 of pregnancy, but results regarding survivability in this study should be considered inconclusive due to low sample size. However, in the companion paper for this study by Halloran et al., it was noted that between breeding and ultrasound pregnancy rates of P4-treated ewes were significantly decreased at 64.7% compared to the 94% pregnancy rates of control ewes. In a previous study of ewes treated with either P4 or CO and necropsied on day 9 or day 12 of pregnancy, ewes received treatments from day 1.5 through the morning of day 9 or day 12 of pregnancy just prior to necropsy [31]. In the present study, P4 therapy was only administered until day 8, therefore the initial increase in P4 concentrations that was observed for P4-treated day 9 ewes was not sustained until day 12. Perhaps, this sudden loss of excess of P4 is the reason for differences in results for likelihood of survival seen in Halloran et al. and suggested in the present study. In contrast, these findings could reflect an adverse effect of P4 treatment to cause an asynchrony between the uterus and conceptus that is known to decrease embryonic survival [50].

Total glucose in uterine flushings increased with advancing day of pregnancy as expected [35] and P4-treated ewes tended to have greater concentrations of glucose in maternal plasma. A key advancement in our knowledge beyond that reported by Satterfield et al. [30] for P4 treatment through day 9 of pregnancy is that there was no effect of P4 treatment on glucose concentrations when pregnancy was extended to day 12 in the present study.

The mRNA expression of two transporters for glucose (*SLC2A1 and SLC5A1*) and one transporter for arginine (*SLC7A1*) were examined in the present study as their expression has been shown to increase in the luteal phase of the estrous cycle and equivalent days of gestation [29] and in response to P4 [30, 47]. Endometrial expression of *SLC2A1*, *SLC5A1*, and *SLC7A1* was affected by day and day × treatment interaction, but not treatment. Endometrial mRNA expression of both glucose transporters (*SLC2A1* and *SLC5A1*) tended to be greater for P4-treated ewes on day 12 of pregnancy which supports previous findings from our laboratory [30].

The significance of the classical (arginine-ornithine-putrescine) and non-classical (arginine-agmatine-putrescine) pathways for synthesis of polyamine is of great interest in our laboratory. The present study is the first to determine whether earlier expression of uterine genes and conceptus development includes increases in expression of *ODC1*, *AZIN2*, and *SLC7A1*. Results of the present study are also the first to indicate that agmatine is very abundant in plasma of sheep and in much greater concentrations than those for putrescine, spermidine,taurine and spermine. Thus, agmatine could act as a major substrate for transport from maternal blood to the conceptus for production of polyamines required for growth and development of conceptuses in sheep. Agmatine plays a vital biological role in the synthesis of polyamines required for growth and development of mammalian conceptuses [41]. In ewes, conceptus development fails when translation of mRNAs for both *AZIN2* and *ODC1* are knocked down since neither arginine nor ornithine can be used for the synthesis of polyamines [44]. In the present study, agmatine was the least abundant polyamine in uterine flushings, but particularly those from P4-treated ewes in which conceptus development was advanced. Perhaps agmatine was used via the novel arginine-agmatine-polyamine pathway to meet demands for polyamines under conditions of accelerated growth and development of the conceptus. Further, concentrations of agmatine may vary depending on whether conceptuses use the classical arginine-ornithine-polyamine pathway or the arginine-agmatine-polyamine pathway as the primary pathway for synthesis of polyamines. It is also possible that the novel arginine-agmatine-polyamine pathway is utilized only when physiological or other conditions, such as nutritional stress, require it to be activated because the classical arginine-ornithine-polyamine pathway is insufficient for the synthesis of required polyamines. It is important to note that concentrations of spermidine tended to be lower in uterine flushings of P4-treated ewes compared to CO-treated ewes as it is the substrate used to produce spermine, perhaps the most bioactive polyamine in the conceptus [51].

Expression of the endometrial cationic amino acid transporter *SLC7A1* mRNA increased significantly between days 9 and 12 of pregnancy in the present study, which is consistent with a reported 4-fold increase in *SLC7A1* between days 10 and 14 of pregnancy [47] in ewes. Previous studies reported an increase in the basic amino acid transporter *SLC7A2B* in endometria of P4-treated day 12 ewes, but there was no difference between day 9 CO-treated and day 9 P4-treated ewes [30]. It is unclear why there was no effect of supplemental P4 to increase expression of *SLC7A1* in the present study, but it could be because *SLC7A1* requires long–term treatment of P4 (20 days) to induce expression of mRNA in uterine LE, GE, and stroma while expression of *SLC7A2* mRNA increased about 4-fold after short-term P4 treatment (10 days) and further increased in response to IFNT [47]. Expression of *AZIN2* mRNA was less in endometria of P4-treated ewes as were concentrations of agmatine and spermidine in uterine flushings of P4-treated ewes. The mechanism responsible for that effect is not known since P4 had been shown to increase expression of *ODC1* in female mice [52] and hamsters [53]. This study has also demonstrated that the protein expression of AGMAT and ODC in the LE is regulated by P4, suggesting that polyamine synthesis may be regulated by sex steroids in the ovine uterus.

In the present study, the most abundant amino acids in maternal plasma were glutamate, glycine, citrulline, arginine, alanine, valine, and leucine. Interestingly, concentrations of aspartate and citrulline were greater in the plasma of P4-treated than CO-treated ewes, we suggest that these two amino acids are precursors for arginine in the pathway responsible for synthesis of NO that increases angiogenesis and vasodilation to increase uterine blood flow during pregnancy [54, 55]. Also, citrulline may be more effective than arginine for maintaining high concentrations of arginine in maternal and fetal blood as there are higher activity levels of arginase in mammalian tissues than enzymes responsible for degradation of citrulline [56]. Glutamate, serine, glycine, and taurine were the most abundant amino acids in uterine flushings in the present study which supports results of a previous study in which glycine, followed by serine, was the most abundant amino acid in uterine flushings from pregnant and cyclic ewes, but were in greater concentrations in pregnant ewes [35]. In contrast, glutamate was in lower concentrations in previous studies, although it was noted to increase 10-fold between days 10 and 14 of pregnancy [35]. Increased concentrations of taurine in uterine flushings from P4-treated ewes was an unexpected finding; however, considering that taurine is a very important antioxidant, osmoregulatory molecule, and regulator of the absorption of lipid soluble vitamins involved in growth and development of the conceptus this perhaps is not unexpected [57]. Concentrations of serine tended to be greater in uterine flushings from P4-treated than CO-treated ewes in the present study suggesting increased substrate for one-carbon metabolism for production of thymidine, purines and *S*-adenosylmethionine, all of which are required for cellular functions supporting growth and development of the conceptus [58]. However, expression of mRNAs for the glycine transporter (*SLC6A9*) and the serine transporter (*SLC1A4*) were greater for CO-treated than P4-treated ewes on day 9 of gestation which is not consistent with no effect of treatment on the abundance of glycine and a slight increase in serine in uterine flushings from P4-treated ewes on day 12 of gestation. The basis for this lack of agreement is not known.

Results of this study confirm that *FGF10* is a key progestamedin during the estrous cycle and early pregnancy [59] and is increased in response to P4 on day 9 of pregnancy [26]. P4-treated ewes had greater expression of *FGF10* than CO-treated ewes on day 9 of gestation, but not on day 12 of pregnancy. We speculate that the exogenous P4 administration increased *FGF10* expression on day 9, which may partially account for accelerated development of ovine conceptuses in ewes treated with P4. Expression of *FGF7* and *HGF* was not affected by treatment of ewes with exogenous P4. *FGF7* is only expressed in blood vessels and is not as highly expressed in ovine stromal cells as *FGF10* [25], therefore, changes in expression of *FGF7* in total homogenized endometrial tissues are difficult to detect. The expression of *HGF* by uterine stromal cells may be constitutive as it was not affected by P4 in the present study or in a previous study in our laboratory [26].

## Conclusions

In conclusion, results of this study indicate that exogenous P4 treatment increased expression of the mitogen *FGF10*, which likely is responsible for the proliferation of trophectoderm cells prior to implantation of the conceptus. Additionally, exogenous P4 treatment decreased endometrial expression of *AZIN2* for synthesis of polyamines, as well as endometrial glycine and serine transporters involved in one carbon metabolism. These findings suggest that while progesterone may accelerate conceptus development, its role in the mechanisms of implantation and pregnancy is complex and warrants further research to investigate its therapeutic properties in livestock reproduction.

## Supplementary Information

**Additional file 1. Table S1.** Primer sequences designed for qPCR analyses.

## Data Availability

The datasets generated in the current study can be made available from the corresponding author upon reasonable request.

## Abbreviations

ANOVA: Analysis of variance
ATP: Adenosine triphosphate
CO: Corn oil
CL: Corpus luteum
Ct: Cycle threshold
GE: Uterine glandular epithelia
HPLC: High performance liquid chromatography
ICM: Inner cell mass
IFNT: Interferon-tau
LE: Uterine luminal epithelial epithelia
LSM: Least square means
NO: Nitric oxide
OPA: *o*-Phthaldialdehyde
RIN: RNA integrity number
P4: Progesterone
SC: Stromal cells
SEM: Standard error of the mean
sGE: Uterine superficial glandular epithelia
Tr: Trophectoderm
qPCR: Quantitative polymerase chain reaction
