# Supplementary material for: Pre-implantation exogenous progesterone and pregnancy in sheep: I. polyamines, nutrient transport, and progestamedins
Source: J Anim Sci Biotechnol. 2021 Mar 5;12:39. doi: 10.1186/s40104-021-00554-6 (PMC7934464; doi:10.1186/s40104-021-00554-6)
Supplement: Supplementary file 1 — Additional file 1. Table S1. Primer sequences designed for qPCR analyses. [file 40104_2021_554_MOESM1_ESM.docx]

Table S1. Primer sequences designed for qPCR analyses

| Gene | Forward primer (5'→3') | Reverse primer (5'→3') |
| --- | --- | --- |
| *SLC2A1* | TGGGAAAGTCCTTTGAGATGC | GGTCAGGCCGCAGTACACA |
| *SLC5A1* | GCTGGAGCCTGCGTAACA | TGAATGTCCTCGTCTTCTGCAT |
| *SLC7A1* | CCTAGCGCTCCTGGTCATCA | GGGCGTCCTTGCCAAGTA |
| *FGF10* | GGAAAACGGATACAATACCTATGCA | TTTCCATTCAATGCCACATACATT |
| *FGF7* | AGTTTGCTCCACAGATCATGCTT | TGCTCTGGAGTCATGTCATTGC |
| *HGF* | ACAGCTTTTTGCCTTCGAGC | AACTCTCCCCATTGCAGGTC |
| *ODC1* | TGCCTTCTATGTTGCGGACC | TGACGGCATAAAAGGGGGTG |
| *AZIN2/ADC* | TCCCTGCCTCTAGAAGCTCACT | ATCGTTTCCACTCCGGATAGAC |
| *AGMAT* | TCTCTTCAAGCTGACCCACCAT | TCACGCCTTCACTGCAGATT |
| *TUB* | GGTCTTCAAGGCTTCTTGGT | CATAATCGACAGAGAGGCGT |
